# Factors effecting on health-promoting behaviors in iranian pregnant women and their husbands: the actor-partner interdependence model (APIM)

**DOI:** 10.1186/s12884-024-06652-3

**Published:** 2024-06-28

**Authors:** Sara Zohouri, Mahbobeh Faramarzi, Reza Ghorban Jahromi

**Affiliations:** 1 Department of Psychology, Science and Research Branch, Islamic Azad University, Tehran, Iran; 2 Population, Family and Spiritual Health Research Center, Health Research Institute , Babol University of Medical Sciences, Babol, Iran; 3 Department of educational and personality psychology, Science and Research Branch, Islamic Azad University, Tehran, Iran

**Keywords:** Expectant couples, Actor-partner interdependence model (APIM), Marital adjustment, Depression, Health-promoting behaviors

## Abstract

**Introduction:**

Pregnancy is an important period of life for women and their husbands as the couple’s health is essential. The present study evaluated the impact of some factors (marital adjustment with depressive symptoms) on health-promoting behaviors in pregnant women and their husbands based on the actor-partner interdependence model (APIM).

**Materials and methods:**

This descriptive study examined 211 couples (pregnant women and their husbands) in pregnancy clinics of Babol University of Medical Sciences using a convenience sampling method. The participants completed Spanier’s Dyadic Adjustment Scale (DAS) (1979), Edinburgh Postnatal Depression Scale (EPDS) (1987), and Walker’s Health Promoting Lifestyle Profile II (HPLPII) questionnaire (1997). The relationships between women and their husbands were also evaluated using structural equation modeling with R software according to the Lavaan (latent variable analysis) package based on APIM-SEM.

**Results:**

The pregnant women’s marital adjustment positively affected their health-promoting behaviors *(β = 0.456*, 95% *Cl*: 0.491–0.998, *p* < 0.001) and their husbands’ (β = 0.210, 95% *Cl*: 0.030–0.726, *p* = 0.048). Pregnant woman’s depressive symptoms also negatively affected their health-promoting behaviors (β=-0.088, 95% *Cl*: -0.974–0.074, *P* = 0.236) and their husbands’ health-promoting behaviors (β=-0.177, 95% *Cl*: -0.281 – -0.975, *P* = 0.011). Furthermore, the husband’s marital adjustment only positively affected his studied behaviors (β = 0.323, 95% *Cl*: 0.0303–0.895, *P* < 0.001) but did not affect the pregnant woman’s health behaviors. The husband’s depressive symptoms had a negative impact on his studied behaviors (β = 0.219, 95% *Cl*: -0.122 – -0.917, *P* = 0.001) and did not affect the pregnant woman’s depressive symptoms. Our findings confirmed the mediating role of depressive symptoms in pregnant women and their husbands on the association of marital adjustment and health-promoting behaviors. According to the actor-partner study, a pregnant woman’s marital adjustment scores positively affected her studied behaviors and her husband (β = 0.071, 95% *Cl*: 0.042–0.278, *P* = 0.015) by decreasing her depression score. Therefore, the husband’s marital adjustment score positively affected his studied behaviors by decreasing his depression score (β = 0.084, 95% *Cl*: -0.053 -0.292, *P* = 0.005), and it did not affect his wife’s health-promoting behaviors.

**Discussion and conclusion:**

These findings suggest healthcare providers, obstetricians, and psychologists evaluate the husbands’ symptoms of depression and health-promoting behaviors in the routine pregnancy care of pregnant women. They also pay great attention to marital adjustment as a determinant of reducing depressive symptoms in pregnant women and their husbands.

**Supplementary Information:**

The online version contains supplementary material available at 10.1186/s12884-024-06652-3.

## Background

Pregnancy causes significant emotional and physical changes [1, 2] and may lead to physical, psychological, and social changes in pregnant women and their husbands through great changes in their feelings and thoughts [3]. Depression is a common mental disorder during pregnancy [4]. The incidence of depressive symptoms is reported to be 10–20% in pregnant women [5]. Based on studies, depressive symptoms are very common in pregnant women, so that one out of every 7 to 10 pregnant women suffers from a common psychiatric disorder [6]. Based on a study in a hospital in Babol County, the prevalence of depressive symptoms is 25% [7]. In addition to mothers, many fathers also experience depression during pregnancy [8]. It is reported that the prevalence of depression is 1–26% in fathers and 24–50% in husbands whose wives are depressed [9].

Marital adjustment is a cause of peace of mind in couples [10]. It refers to a state in which a woman and her husband have a general feeling of satisfaction and happiness about their marriage and each other. Marital adjustment is a constant and changing course. It also does not refer to the absence of problems in life but to the capacity to adapt and solve problems [11–13]. Marital adjustment can be considered a psychological situation that causes destructive effects on a couple’s physical and mental health [14]. Evidence indicates that marital adjustment affects depression, so women experience big worries during this period and become depressed about adjusting to the situation [15]. The results of a study on 26 Indian couples indicated a significant relationship between marital adjustment and depression [16]. Similarly, a study on 450 Turkish fathers in 2019 reported that fathers with satisfactory marital relationships were less at risk of depression [17].

Health-promoting behaviors, which cover social relationships, health responsibilities, self-actualization, stress management, nutrition, and physical activity, directly link disease prevention by maintaining or increasing health and self-efficacy [18]. Health-promoting behaviors can be regarded as a way to achieve a better quality of life by coping with and adapting to psychological problems and stresses and improving interpersonal relationships [19]. Given the significant importance of such behaviors during pregnancy, it is vital and necessary to pay attention to and strengthen them during this period [18]. Studies also support the effects of depression on an individual’s health-promoting behaviors. According to a study of 1949 people in China in 2022, the more health behaviors are promoted, the less likely depressive symptoms will occur [20].

In the current study, the Actor-Partner Interdependence Model (APIM) was employed to assess the mutual impact of the characteristics of each spouse. For the analysis of couples’ data in APIM, it is believed that people are affected not only by their characteristics but also by their spouses’ characteristics. Therefore, the analysis is not focused on the person. Still, the couple is the unit of analysis in such a way that both the effects of the characteristics of each couple on their predictor variables (actor effect) and the effects of the characteristics of each couple on the predictor variable of their spouses (partner effect) are evaluated [21]. A study on 141 couples in Tehran using the APIM indicated that dyadic satisfaction affected their depression. Furthermore, men’s dyadic satisfaction affected their wives’ depression. In other words, the actor effect was significant for men and women, and the partner effect on men’s satisfaction was significant [22].

According to the content above, evidence suggests that marital adjustment affects depression in women. Moreover, depression can affect the couple’s health behaviors. However, depression’s mediating role between marital adjustment and health-promoting behaviors is unknown. The effect of marital adjustment contrast in women and men on their depressive symptoms or health behaviors is also unknown. In order to fill the information gap, the present study evaluated the association of marital adjustment with depressive symptoms and health-promoting behaviors in pregnant women and their husbands using the actor-partner interdependence model (APIM). Based on conceptual model (Supplementary Fig S1), this study suggests the following hypotheses: (1) Pregnant women’s marital adjustment affects their health-promoting behaviors besides their husbands’. (2) Husbands’ marital adjustment affects their health-promoting behaviors besides those of their wives.3) Pregnant women’s marital adjustment affects depressive symptoms of themselves and their husbands. 4) Husbands’ marital adjustment affects depressive symptoms of themselves and pregnant women. 5) Pregnant women’s depressive symptoms affect their health-promoting behaviors as well as their husbands’. 6) Husbands’ depressive symptoms affect their health-promoting behaviors as well as their pregnant wives’. 7) Pregnant women’s depressive symptoms mediate between health-promoting behaviors and marital adjustment. 8) Husbands’ depressive symptoms mediate between health-promoting behaviors and marital adjustment.

## Methods

The present descriptive-analytical research had a correlational type. Its statistical population included pregnant women and their husbands who visited two pregnancy clinics of teaching hospitals (Ayatollah Rouhani and Yahyanejad Hospitals) and health centers of Babol University of Medical Sciences from May to September 2022. The Ethics Committee of Islamic Azad University, Science and Research Branch (IR.IAU.SRB.REC.1401.090) approved this research. All participants provided written informed consent before inclusion in the study. The inclusion criteria were pregnant women who was married and living with their husband, aged over 18 years, gestational age over 13 weeks, self and her husband’s consent to be included in the study, and education level of higher than primary school for herself and her husband. Pregnant women who did not live with their husband were excluded. Also, pregnant women who lived with a partner (not legally married) were excluded. Furthermore, convenience sampling was performed. The sample size was estimated 211 with respect to 0.27 ratio of health-promotion behaviours in the pilot study before the start of the study, α = 0.05, and d = 0.06.

A midwife knowledgeable about the study invited eligible pregnant women to enter the study during prenatal visits in obstetric clinics. If the pregnant woman and her husband met the primary eligibility requirements (married subjects and consent to enter the study), they were referred to the researcher, the first author (S.Z). The researcher contacted the couples by phone, checked the inclusion/exclusion criteria using phone interviews with men and women separately, and then explained the research objectives. She gave explanation regarding the purpose of the study and how to fill in the questionnaires. The researcher asked the women and men to fill out separately the questionnaires. Then, she sent separately to men and women the online link of the questionnaires (via the DigiSurvey^®^ platform) via messages to complete at home, within a week or less.

In this study, 300 couples were referred to the first author by midwives. Among them, 32 couples did not answer the researcher’s phone call or refused to be included in the study after answering. Furthermore, 268 couples agreed to complete the questionnaires. A total of 536 questionnaires were collected, which left 422 after removing incomplete questionnaires. Finally, 211 couples completed all research questionnaires, including Spanier’s Dyadic Adjustment Scale (DAS), Walker’s Health Promoting Lifestyle Profile II (HPLPII) questionnaire, Edinburgh Postnatal Depression Scale (EPDS), and demographic questionnaires, and their information was inserted into the software.

The participants were talked to, and the research purpose was explained before sending the link to the questionnaires to the participants online or giving the questionnaires in person. If the couples had consent to cooperate, they received the questionnaires. Once questionnaires were collected, the data of 211 couples were inserted into SPSS.

### Questionnaires

Age, education level, and job status options were similar in the Demographic questionnaires for women and men, but information about pregnancy was added to the women’s Demographic questionnaire.

Spanier’s Dyadic Adjustment Scale (DAS) was developed in 1976 by Graham B. Spanier at The Pennsylvania State University as a self-report measure of the quality of a marriage or similar relationship [23]. It includes 32 questions for assessing the quality of a relationship from the spouses’ viewpoint. The items are on a Likert scale with a score of 0 to 151. Questions 1–22, 25–28, and 32 are scored on a scale of 0 to 5, 23 and 24 on a scale of 0 to 4, 29 and 30 on a scale of 0 to 1, and 31 on a scale of 0 to 6. Higher scores indicate greater dyadic adjustment. The scale assesses four characteristics: cohesion, dyadic satisfaction, affection expression, and consensus. The Persian DAS reliability was 0.91 [24]. Earlier research in an Iranian population employed the Persian DAS [25]. In Iran, this scale was translated, implemented and standardized by Amozgar and Hossein Nejad in 1995 [26]. Cronbach’s alpha of 0.89 was used in our study to determine the reliability of DAS.

In order to measure depression, the Edinburgh Postnatal Depression Scale (EPDS), with ten four-choice questions, was used. of each question receive a score from zero to three on the basis of the sign severity, and the score a person receives is calculated by adding the scores of 10 questions, with a range from 0 to 30. By calculating the simultaneous correlation coefficients of the EPDS and the Beck Depression Inventory (BDI), the test validity was assessed to be 0.87, and its reliability was evaluated to be 0.75 using Cronbach’s alpha and the split-half approach [27]. In Iran, the sensitivity and specificity of this questionnaire were obtained as 95.3% and 87.9%, respectively. Cronbach’s alpha coefficient was obtained as 0.83 for the scale [28].

Health-Promoting Lifestyle Profile II (HPLPII) questionnaire [29] has 52 items with six subscales: health responsibility, nutrition, self-actualization, stress management, physical activity, and interpersonal relationships. The choices are on a 4-point Likert scale, specifically never = 1, sometimes = 2, often = 3, and always = 4. The total health-promoting lifestyle score ranges from 52 to 208. Using Cronbach’s alpha, its reliability was 0.86 for self-actualization, 0.85 for physical activity, 0.86 for health responsibility, 0.79 for stress management, 0.87 for interpersonal relationships, and 0.80 for nutrition. For six dimensions, the range was 0.79 to 0.86, and for the whole questionnaire, it was 0.94 [29]. The Persian version’s reliability was 0.81 for the whole questionnaire according to Cronbach’s alpha [30].

### Analysis

Descriptive and demographic characteristics of the research were evaluated using SPSS-26.

to find the status of the samples for each variable. This software also turned the data file structure from an individual to a dyadic structure. APIM was utilized to explain the couple’s interdependence regarding the intra- and interpersonal nature of depressive symptoms, health-promoting behaviors, and marital adjustment. APIM aims to investigate the relationship between each person’s marital adjustment with depressive symptoms and health-promoting behaviors, as well as each person’s depressive symptoms with health-promoting behaviors (actor effect) and each person’s marital adjustment with spouse’s depressive symptoms and health-promoting behaviors, as well as each person’s depressive symptoms with spouse’s health-promoting behaviors (partner effect) [31]. Structural equation modeling (SEM) was used to test the main model since it establishes a correlation between the measurement error values, as the correlation between the errors of the dependent variables was a sign of the relationship between the criterion variable scores of the spouses. This method considers the interdependence of dyadic data. Furthermore, the condition of interdependence in linear regression models is not established.

**Fig. 1:**
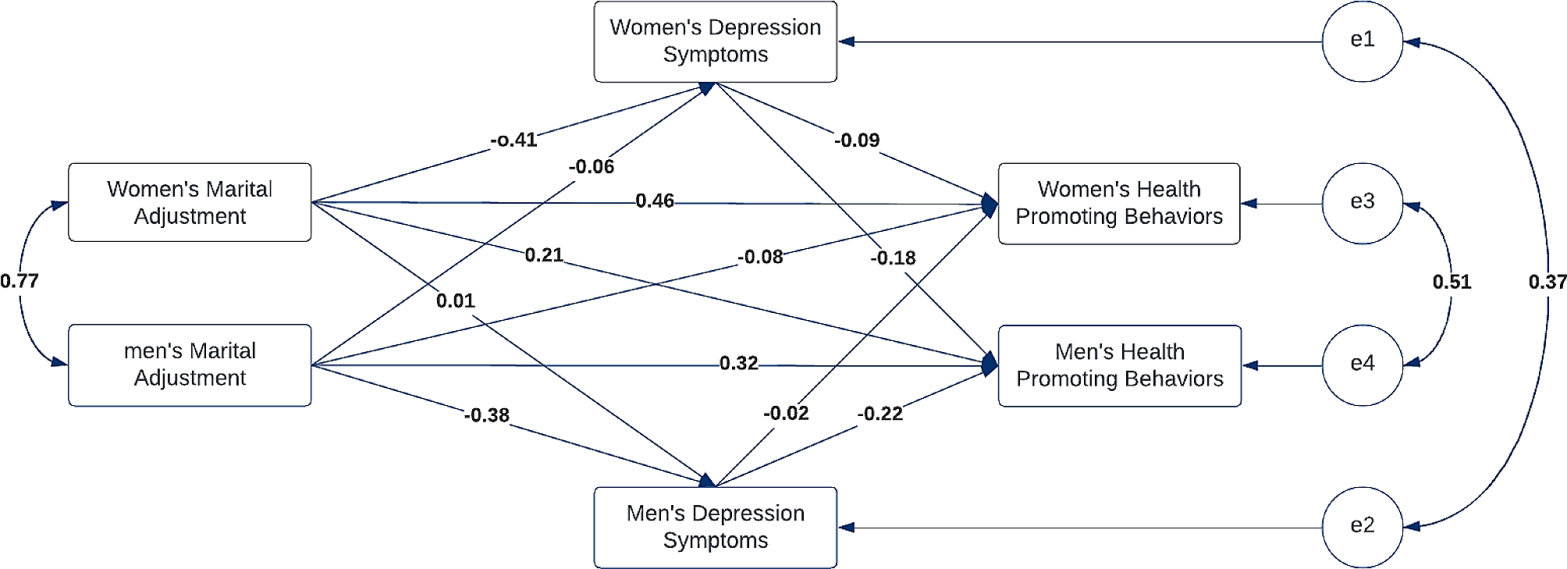
Final actor-partner interdependence model (APIM) of the effect of marital adjustment and depressive symptoms on health-promoting behaviors in pregnant women and their husbands

To this end, the research hypotheses were evaluated using R software and the Lavaan package according to the APIM-SEM package [32]. Lavaan stands for Latent Variable Analysis and provides researchers with tools to explore, evaluate, and comprehend a wide group of latent variable models consisting of factor analysis and structural equations (32). Also, based on Kenyy (2015) great effort has been undertaken to ensure the accuracy of the APIM-SEM [33]. *Β* Coefficients of this study (Tables 1 and 2; Fig. 1) shows that accuracy of APIM is suitable for explanation of the effect of marital adjustment and depressive symptoms on health-promoting behaviors in pregnant women and their husbands.

**Table 1:** APIM analysis for direct effects of marital adjustment and depression among pregnant women and their husbands

Gender			Estimate	Confidence Interval 95%	β	*P* value
Women	Marital adjustment → Health-Promoting Behavior	Actor	0.746	0.491–0.998	0.456	< 0.001
		Partner	0.394	0.030–0.726	0.210	0.048
	Marital adjustment → Depression symproms	Actor	-0.138	-0.073 – -0.201	-0.405	< 0.001
		Partner	0.002	-0.056–0.062	0.008	0.935
	Depression symptoms → Health-Promoting Behavior	Actor	-0.424	-0.974–0.074	-0.088	0.236
		Partner	-0.774	-0.281 – -0.975	-0.177	0.011
Men	Marital adjustment → Health-Promoting Behavior	Actor	0.576	0.0303–0.895	0.323	< 0.001
		Partner	-0.126	-0.379–0.123	-0.081	0.414
	Marital adjustment → Depression symproms	Actor	-0.110	-0.052 – -0.168	-0.381	< 0.001
		Partner	-0.019	-0.079–0.046	-0.059	0.537
	Depression symptoms → Health-Promoting Behavior	Actor	-0.357	-0.122 – -0.917	-0.219	0.001
		Partner	-0.086	-0.668–0.592	-0.016	0.823

Table 2 presents the indirect impacts of the model with depression scores’ mediating role also, Fig. 1 presents the final actor-partner interdependence model (APIM) of the effect of marital adjustment and depressive symptoms on health-promoting behaviors in pregnant women and their husbands. The results showed that pregnant women’s depressive symptoms had a mediating role between martial adjustment and their health- behaviors (β = 0.035, 95% *cl*: 0.002–0.159 promoting, *P* = 0.046,) (actor-actor-actor). Furthermore, pregnant women’s depressive symptoms had a mediating role between husbands’ health-promoting behaviors and marital adjustment (β=-0.071, 95% *cl*: 0.278 − 0.042, *P* = 0.015); hence, the actor-actor-partner effect was significant. However, men’s depressive symptoms had no mediating role between pregnant women’s marital adjustment scores with their health-promoting behaviors (β=-0.001, 95% *Cl*: -0.025-0.021, *P* = 0.920) (actor-partner-actor) or their husbands (β=-0.002, 95% *Cl*:-0.093-0.073, *P* = 0.907) (actor-partner-partner). Husbands’ depressive symptoms only mediated to marital adjustment and their studied behaviors (β = 0.084, 95% *cl*: 0.053–0.292 *P* = 0.005) (actor-actor-actor). Mediating roles of males’ depressive symptoms in pregnant females’ marital adjustment and studied behaviors were not confirmed (β = 0.005, 95% *Cl*: -0.010-0.061, *P* = 0.363,) (actor-actor-partner)

**Table 2:** APIM analysis results for indirect effect

Indirect paths	Estimate	Confidence Interval 95%	β	*P* value
FMA → FD→ FHPB	0.058	0.002–0.159	0.035	0.046
FMA → FD→ MHPB	0.134	0.042–0.278	0.071	0.015
FMA → MD→FHPB	-0.001	-0.025 -0.021	-0.001	0.920
FMA → MD → MHPB	-0.003	-0.093 -0.073	-0.002	0.907
MMA → FD → FHPB	0.008	− 0.0.010–0.061	0.005	0.363
MMA → FM → MHPB	0.019	-0.031 -0.104	0.011	0.438
MMA →MD → FHBP	0.009	-0.067 -0.078	0.006	0.836
MMA →MD → MHPB	0.149	-0.053 -0.292	0.084	0.005

*FMA: Female’s Marital Adjustment, FD: Female’s Depression symptom, FHPB: Female’s Health Promoting Behavior, MMA: Male’s Marital Adjustment, MD: Male’s Depression symptom, MHPB: Male’s Health Promoting Behavior

## Results

Table 3 presents the demographic characteristics of pregnant women and their husbands. The women’s mean gestational age was 29.69 ± 5.99, and more than half of the women and their husbands were 25–30 years old. The husbands’ mean average age was 33.55 ± 5.42. Most men were employed, but only 18.5% of women were employed.

**Table 3:** Demographic characteristics of pregnant women and their husbands (*n* = 211)

Demographic variable	Level	pregnant women		Husbands	
		**Number**	**Percentage**	**Number**	**Percentage**
Age (year)	18–24	60	28.4	10	4.7
	25–35	111	52.6	131	62.1
	> 36	40	19	70	33.2
Job	Unemployed	172	81.5	-	-
	Employee	39	18.5	211	100
Education level	Primary school	22	10.5	47	22.3
	High school	87	41.2	72	34.1
	University	102	48.3	94	43.6

According to Table 4 and the t-test of correlated samples, no significant difference was found between the mean total score of marital adjustment and its sub-components in women and men (*P* > 0.05). The mean of depression symptoms in women was significantly higher than in men (*P* < 0.001). The health-promoting behaviors’ total score was meaningfully higher in women compared to in men (*P* < 0.001). Still, no significant difference was observed between men and women in nutrition scores and interpersonal relationships (*P* > 0.05).

**Table 4:** Descriptive indicators and the difference between the averages of men and women

Variable	Pregnant women Mean	Men Mean	Difference in means	T	*P* value
Marital Adjustment	112.54(15.06)	112.40(15.84)	0.142	1.195	0.846
Marital satisfaction	38.79(3.76)	38.72(4.00)	0.061	0.230	0.818
Reciprocal correlation	17.18(3.48)	17.20(3.62)	-0.018	-0.096	0.923
Reciprocal agreement	47.54(9.14)	47.57(9.67)	-0.033	-0.075	0.940
Affective expression	9.02(1.57)	8.89(1.65)	0.132	1.296	0.196
Depression Symptoms	8.92(5.12)	7.10(4.57)	1.815	5.132	< 0.001
Health-Promoting Behavior	149.76(24.63)	143.57(28.28)	6.194	3.592	< 0.001
Nutrition	34.28(5.91)	34.91(6.18)	-0.630	-1.564	0.119
Physical activities	38.13(7.38)	33.85(8.60)	4.274	7.264	< 0.001
Health Responsibility	23.48(4.39)	22.75(4.76)	0.729	0.363	0.019
Stress Management	15.19(3.51)	14.25(3.72)	0.905	3.320	0.001
Interpersonal support	16.19(6.08)	16.83(7.13)	-0.639	-1.202	0.201
Self-Actualization	22.46(4.64)	20.91(4.82)	1.554	4.753	< 0.001

The results of Table 1 confirm the first hypothesis in such a way that marital adjustment in pregnant women had significant positive effects on their studied behaviors (actor effect) (β = 0.456, 95% *Cl*: 0.491–0.998, *P* < 0.001) and their husbands’ (partner effect) (β = 0.210, 95% *Cl*: 0.030–0.726, *P* = 0.048).Also, Husbands’ marital adjustment had a substantial positive impact on their studied behaviors (actor effect) (β = 0.323, 95% *Cl*: 0.0303–0.895, *P* < 0.001) but no effect on their pregnant wives (partner effect) (β=-0.081, 95% *Cl*: -0.379–0.123, *P* = 0.414). The pregnant women’s marital adjustment scores had a negative effect on their depressive symptoms (actor effect) (β=-405.0, 95% *Cl*: -0.073 – -0.201, *P* < 0.001) but no effect on husbands’ depressive symptoms (partner effect) (β = 0.008, 95% *Cl*: -0.056–0.062, *P* = 0.935). The husbands’ marital adjustment scores had a negative effect on their depressive symptoms (actor effect) (β= -0.381, 95% *Cl*: -0.052 – -0.168, *P* < 0.001), but they had no significant effect on pregnant women’s depression (partner effect) (β=-0.059, 95% *Cl*: -0.079–0.046, *P* = 0.537). The women’s depressive symptom scores did not affect their health-promoting behaviors (actor effect) (β=-0.088, 95% *Cl*: -0.974–0.074, *P* = 0.236) but significantly impacted their husbands’ (partner effect) (β=-0.177, 95% *Cl*: -0.281 – -0.975, *P* = 0.011). The husbands’ depressive symptoms negatively affect their health-promoting behaviors scores (actor effect) (β=-0.219, 95% *Cl*: -0.122 – -0.917, *P* = 0.001,), but not those of their wives (partner effect) (β=-0.016, 95% *Cl*: -0.668–0.592, *P* = 0.823).

## Discussion

The study confirmed that the pregnant woman’s marital adjustment level positively affected her and her husband’s health-promoting behaviors. A pregnant woman’s depressive symptoms negatively affected her and her husband’s health-promoting behaviors. Consistent with these results, good marital relationships, proper participation in financial and familial issues, and dyadic satisfaction improved and prevented depression among couples [34]. A study in India indicated a high negative correlation between depression and marital adjustment [35]. Another research in Turkey reported that as women’s health status decreased, the risk of depression increased and marital adjustment decreased [36]. A study in Korea also reported that improving marital adjustment was effective in enhancing the health-promoting behaviors of couples during pregnancy [37].

Our results indicated that the husband’s marital adjustment only positively affected his health-promoting behaviors but did not affect the pregnant woman’s health behaviors. Furthermore, the husbands’ depressive symptoms negatively affect their health-promoting behaviors but not those of their wives. Consistent with these results, a study in Korea indicated that men with low marital adjustment were more likely to use alcohol and drugs such as cigarettes as unhealthy behaviors than those with high marital adjustment [38]. Based on evidence, increasing the time and intensity of physical activity in men caused a further reduction in the prevalence of depression in them and increased mental health [39].

Our findings confirmed the mediating roles of depressive symptoms in pregnant women and their husbands between marital adjustment and health-promoting behaviors. Consistent with these findings, previous studies indicated that reducing negative couple emotions in stressful situations predicted marital adjustment. Furthermore, stress management was a component of health-promoting behaviors. Previous studies also reported that each couple’s marital adjustment was associated with higher dyadic satisfaction [13].

The findings of the actor-partner effect also confirmed that the pregnant woman’s marital adjustment scores positively affected the health-promoting behaviors of herself and her husband by decreasing the woman’s depression score. However, the husband’s marital adjustment score only had a positive impact on his studied behaviors by reducing his depression score, but it was not effective in improving health-promoting behaviors. Our results were consistent with Rao’s findings [16], who reported a significant relationship between marital adjustment and depression. Studies collected from review research indicate that the lack of dyadic satisfaction is a risk factor for a father’s depression [40].

Finally, the important question is why pregnant women’s marital adjustment affects their and their husbands’ health behaviors and depression. Still, husbands’ marital adjustment only affects their health behaviors and depression and does not affect the pregnant woman. In other words, spouses only play the actor roles, and their partner’s roles are not confirmed in the model, but pregnant women play both actor and actor-partner roles in important aspects of the model. The answers to these questions are still unclear due to a few numbers of studies. However, there are several assumptions; first, evidence indicates that men care less for their health than women. Based on an analysis in America, men visit primary healthcare centers and diagnostic services much less than women, so the annual consumption of healthcare costs is much higher in women than in men [41]. Second, evidence confirms that women can persuade men to attend health centers. A study in Los Angeles reported that women significantly affected men’s decisions to visit medical care centers [42].

These results have many clinical uses for family specialists, psychiatrists, obstetricians, gynecologists, and healthcare providers. Our study suggests family specialists pay special attention to marital adjustment as a determinant of health promotion in pregnant women and their husbands. Our findings also suggest psychologists pay special attention to the unfavorable adverse effects of depression on health behaviors and identify and treat depression in pregnant women and their husbands. These results also suggest obstetricians and gynecologists pay more attention to husbands’ mental health and health behaviors in the routine pregnancy care of pregnant women because the health behavior scores are lower in men than in women.

In spite of the unique results of the present study, it also had some limitations; first, this was a cross-sectional study. Second, these results were conducted in a city and academic center; hence, the results cannot be generalized. Third, the results and dyadic relationships are affected by social and cultural factors of society where the couple has grown up. Therefore, future studies should be conducted with larger sample sizes and multinational nature to investigate the effects of dyadic relationships and their relationship with health behaviors based on the cultural context of society.

## Conclusion

The study confirmed the mediating roles of depressive symptoms in pregnant women and their husbands between marital adjustment and health-promoting behaviors. The actor-partner effect also confirmed that the pregnant woman’s marital adjustment scores positively affected the health-promoting behaviors of herself and her husband by decreasing the woman’s depression score. However, the husband’s marital adjustment score only had a positive impact on his studied behaviors by reducing his depression score, but it was not effective in improving health-promoting behaviors.These findings suggest healthcare providers, obstetricians, and psychologists pay more attention to husbands’ mental health and health behaviors in routine pregnancy care for pregnant women.

### Electronic supplementary material

Below is the link to the electronic supplementary material.

Supplementary Material 1

## Data Availability

The data that support the findings of this study are available from the correspondingauthor upon reasonable request.

## Abbreviations

APIM: Actor-Partner Interdependence Model
DAS: Dyadic Adjustment Scale
EPDS: Edinburgh Postnatal Depression Scale
HPLPII: Walker’s Health Promoting Lifestyle Profile II
BDI: Beck Depression Inventory
SEM: Structural equation modeling
