# Supplementary material for: Factors effecting on health-promoting behaviors in iranian pregnant women and their husbands: the actor-partner interdependence model (APIM)
Source: BMC Pregnancy Childbirth. 2024 Jun 28;24:450. doi: 10.1186/s12884-024-06652-3 (PMC11214222; doi:10.1186/s12884-024-06652-3)
Supplement: Supplementary file 1 — Supplementary Material 1 [file 12884_2024_6652_MOESM1_ESM.docx]

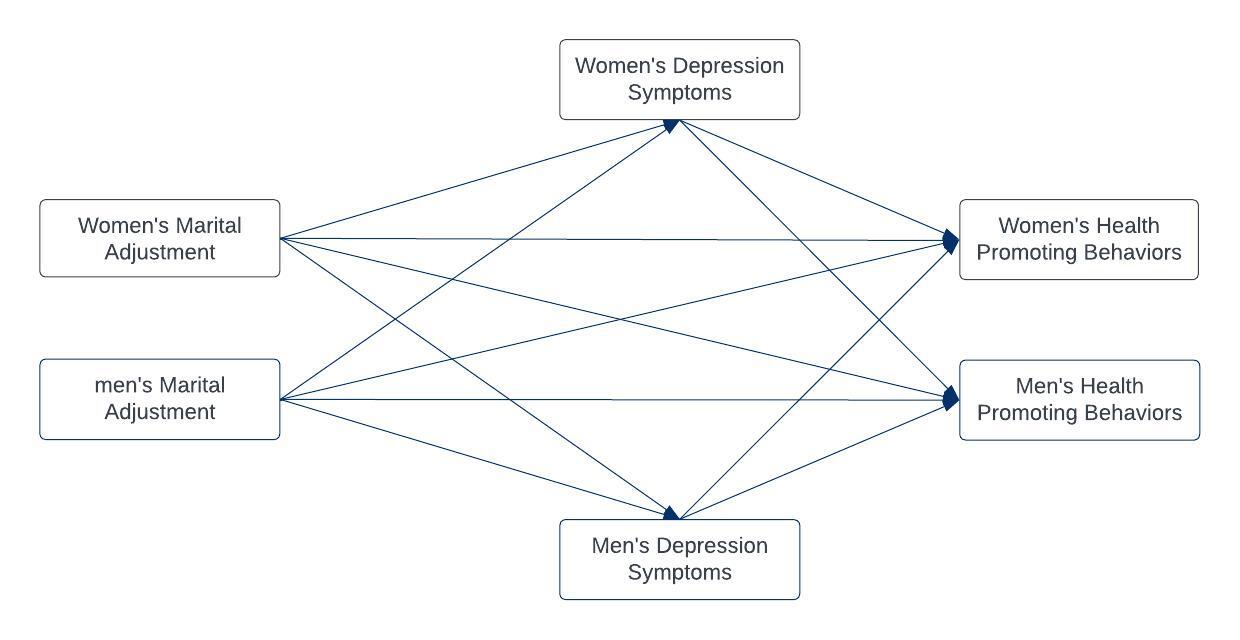


Figure S1. Conceptual model hypothesizing of the effect of marital adjustment and depressive symptoms on health-promoting behaviors in pregnant women and their husbands
